# Solid and Cystic Hidradenoma of the Forearm: A Case Report

**DOI:** 10.7759/cureus.114174

**Published:** 2026-08-08

**Authors:** Katherine Rivero, Lilly Xie, Susan Chung

**Affiliations:** 1 General Surgery, BronxCare Health System, Icahn School of Medicine at Mount Sinai, Bronx, USA; 2 Plastic Surgery, Bronx-Lebanon Hospital Center, Bronx, USA; 3 Plastic and Reconstructive Surgery, BronxCare Health System, Icahn School of Medicine at Mount Sinai, Bronx, USA

**Keywords:** case report, cutaneous neoplasm, forearm mass, hidradenoma, sweat gland tumor

## Abstract

A 41-year-old woman presented with a two-year history of a gradually enlarging, tender nodule on the left forearm. Ultrasonography revealed a subcutaneous mass with both solid and cystic components. The lesion was surgically excised in its entirety with local flap closure, and the postoperative course was uneventful. Pathological evaluation confirmed a solid-cystic hidradenoma, a rare tumor of sweat gland origin. While the coexistence of solid and cystic components is an inherent, defining feature of this tumor subtype rather than an atypical finding, this case is notable for its occurrence on the volar forearm, a site with very few comparable adult cases in the published literature. Because hidradenoma is easily mistaken for more common and, in the case of dermatofibrosarcoma protuberans, potentially malignant, cutaneous lesions, recognizing this diagnosis at atypical sites has direct implications for surgical planning and patient counseling. We present this case to contribute to the limited literature on adult extremity hidradenomas and to reinforce the value of including hidradenoma in the differential diagnosis of nodular cutaneous lesions arising outside its typical head, neck, and trunk distribution.

## Introduction

Solid and cystic hidradenoma is a rare, benign adnexal tumor derived from either the eccrine or apocrine sweat glands [1]. These tumors are most commonly reported on the head, neck, or trunk, with occurrence in the extremities considered uncommon [1,2]. As a result, hidradenomas are frequently misdiagnosed as more common skin conditions, such as epidermoid inclusion cysts or dermatofibromas. Hidradenomas also lack a distinctive clinical presentation, further complicating diagnosis and presenting a diagnostic challenge [3]. This case is notable primarily for its atypical anatomical location, since the solid and cystic morphology described below is an inherent feature of this tumor subtype rather than an atypical one. Recognizing hidradenoma as a differential diagnosis outside its typical distribution carries direct clinical relevance, since lesions at extremity sites are more likely to be mistaken for other entities, including dermatofibrosarcoma protuberans, a locally aggressive tumor with a very different management pathway, and may otherwise face diagnostic delay.

## Case presentation

A 41-year-old, right-hand-dominant woman with a history of asthma presented with a nodular lesion on her left forearm that had been slowly enlarging over more than two years. She denied preceding trauma, numbness, tingling, or drainage. Physical examination revealed a nodular lesion measuring 3 x 2 cm on the radial aspect of the left volar forearm (Figure 1). The lesion was mobile with irregular nodularity and tender to palpation. Initial clinical differential diagnoses included epidermal inclusion cyst, pilomatricoma, dermatofibroma, and dermatofibrosarcoma protuberans; hidradenoma was not considered at this stage.

**Figure 1:**
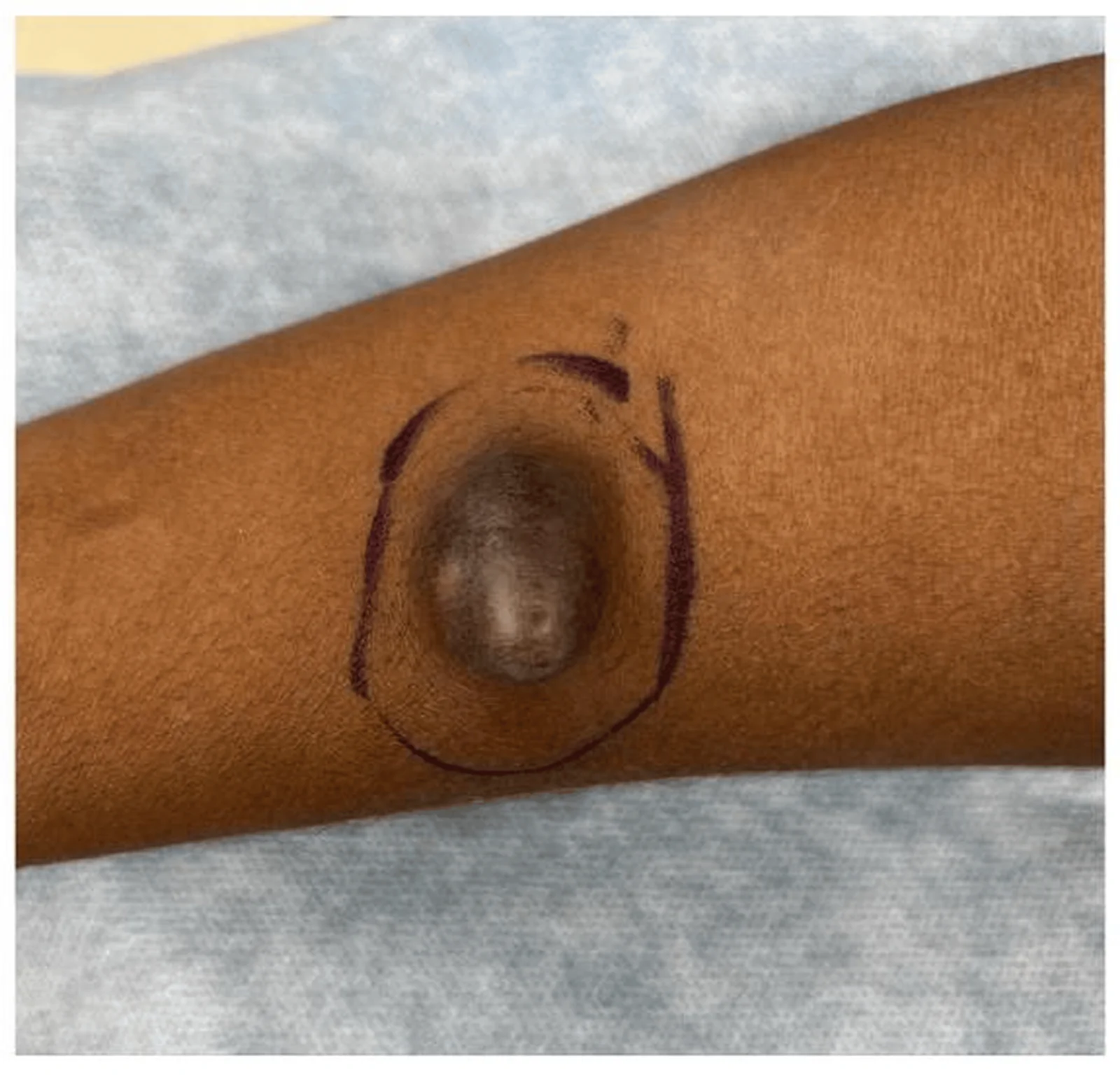
The nodule on the radial aspect of the left volar forearm is 3 x 2 cm in size, well-circumscribed and raised.

Ultrasonography was obtained for better characterization of the lesion given its proximity to important surrounding anatomical structures. Imaging revealed a subcutaneous mass of mixed echogenicity measuring 3.6 x 2.0 x 1.4 cm (Figure 2), with both cystic and solid elements but an otherwise indeterminate appearance.

**Figure 2:**
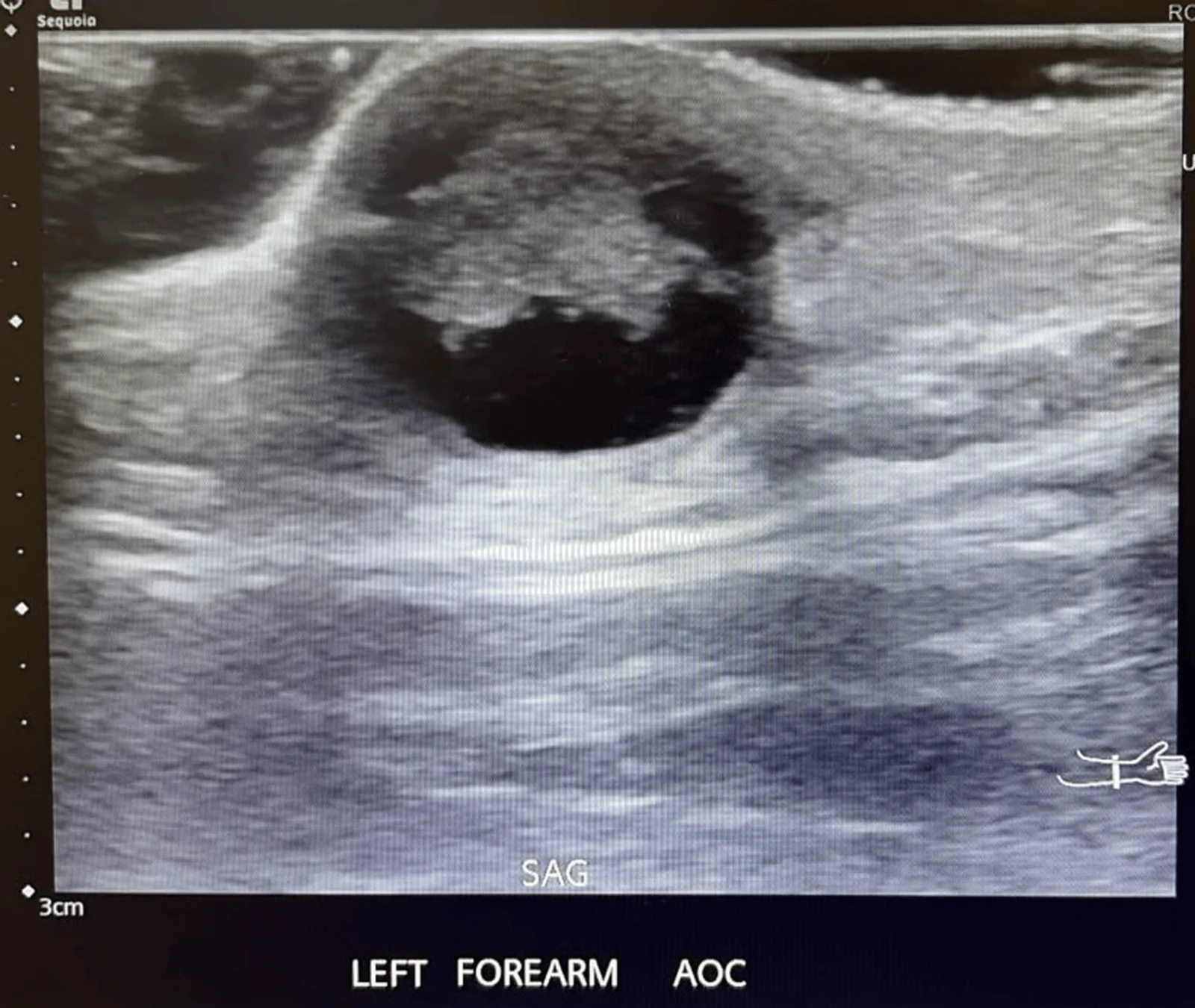
A subcutaneous mass of mixed echogenicity measuring 3.6 x 2.0 x 1.4 cm is noted on ultrasonography.

CT with contrast and MRI were considered for further characterization prior to excision; however, the patient elected to proceed without additional preoperative imaging.

The patient underwent surgical excision under local anesthesia. The mass was separated from the surrounding subcutaneous tissue and excised in its entirety, then sent for pathological analysis. Given the size of the defect, a local flap was transposed to achieve closure. The immediate postoperative recovery was uneventful, with no early wound complications.

The excision was limited to the subcutaneous plane, and the deep fascia was not taken. No predetermined margin of normal tissue was planned; rather, the mass was excised in its entirety, corresponding to an approximately 4 x 3 cm specimen intraoperatively. On pathologic assessment, the closest margin was 2 mm (0.2 cm).

The specimen was received in formalin, labeled "left forearm lesion," and consisted of an unoriented oval piece of skin measuring 2.5 x 2.3 cm with attached subcutaneous tissue measuring 3.0 x 2.5 x 1.5 cm (Figure 3). The skin contained a raised, soft nodule measuring 1.2 x 1.0 x 0.8 cm, located 0.2 cm from the nearest margin; the remaining skin was unremarkable. The margin was inked green, and sectioning revealed a 2.3 x 2.0 x 1.5 cm unilocular cystic cavity filled with a yellow, semi-translucent, sticky material. Representative sections were submitted in two blocks.

**Figure 3:**
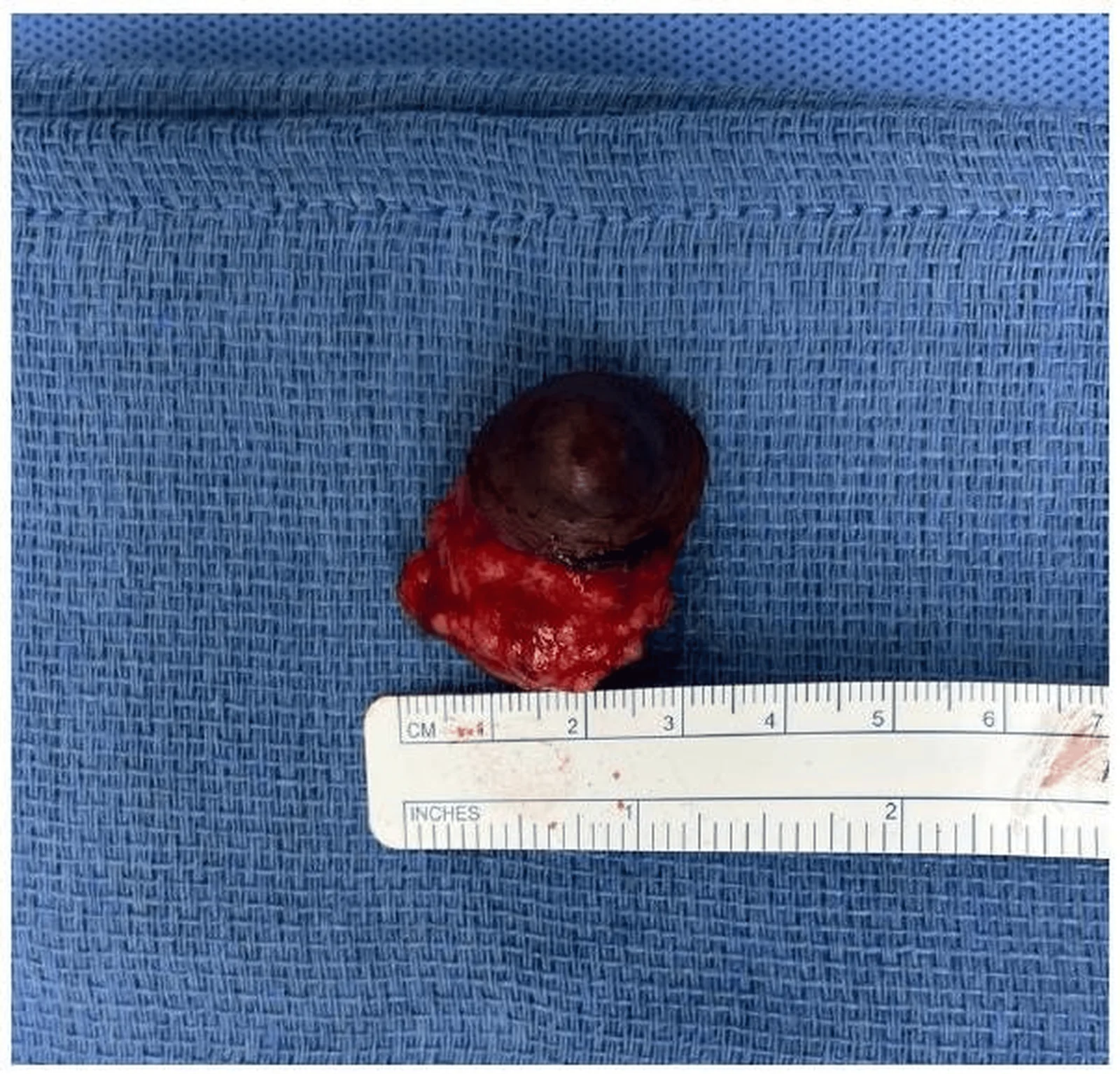
The excised specimen measuring 2.5 x 2.3 cm, with attached subcutaneous tissue measuring 3.0 x 2.5 x 1.5 cm.

To clarify the relationship between the measurements obtained at each stage of evaluation: the 3 x 2 cm dimension recorded on physical examination corresponds to the palpable cutaneous and superficial subcutaneous extent of the lesion, while the 3.6 x 2.0 x 1.4 cm dimension on ultrasonography reflects the full subcutaneous extent of the mass, including deeper tissue not fully appreciable on palpation. On gross sectioning, this mass was found to comprise two components: a raised nodule (1.2 x 1.0 x 0.8 cm) corresponding to the more superficial, palpable portion, and a separate unilocular cystic cavity (2.3 x 2.0 x 1.5 cm) representing the deeper, fluid-filled component that contributed to the larger overall dimensions seen on ultrasonography.

Histological examination revealed solid and cystic components at low-power magnification (Figure 4). At higher power, the solid areas were composed predominantly of polygonal cells with eosinophilic cytoplasm, and clear cell change was also observed (Figure 5). 

**Figure 4:**
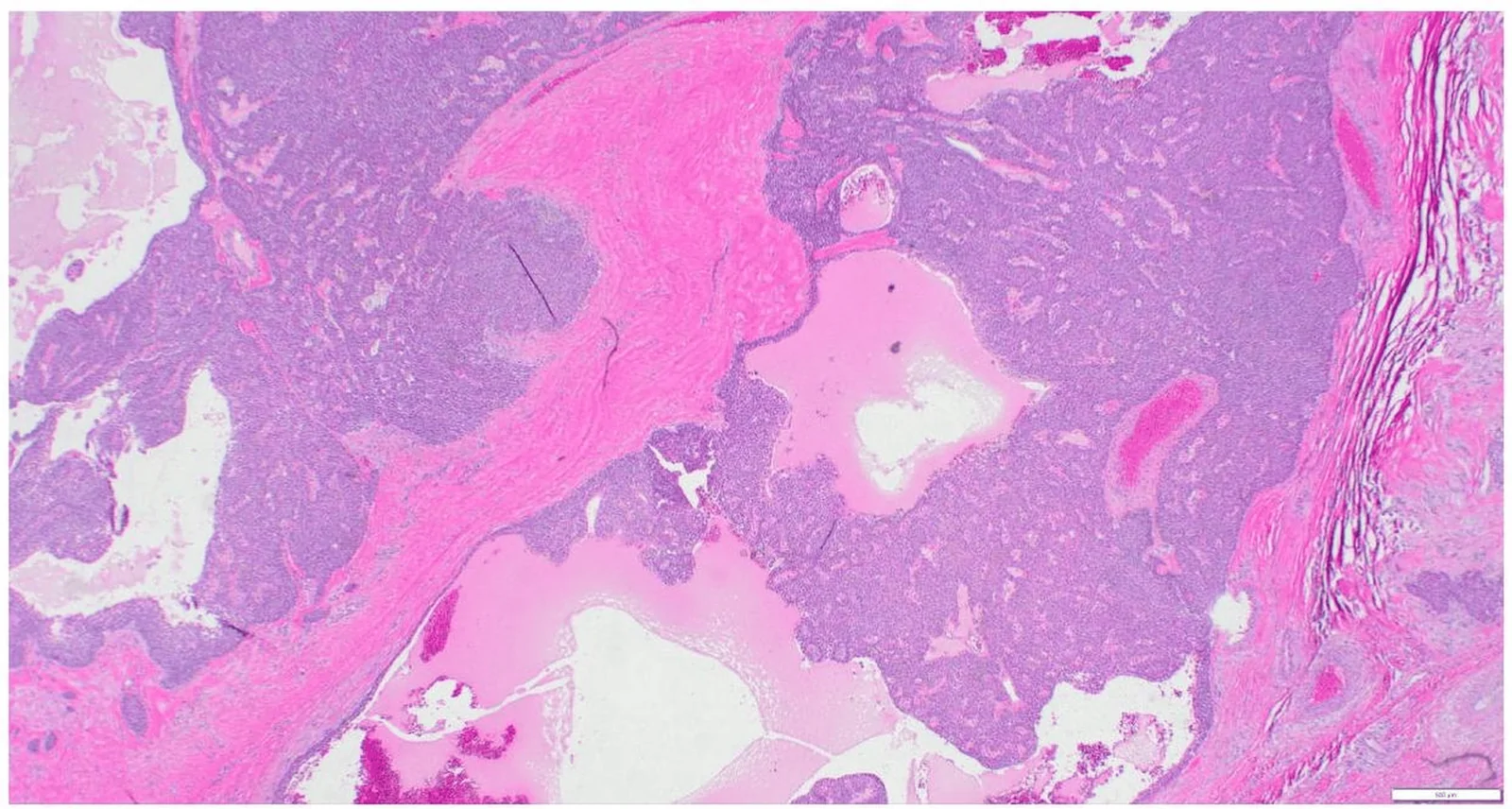
Low-power magnification of the lesion demonstrates solid and cystic areas. (H&E, scale bar = 500 μm).

**Figure 5:**
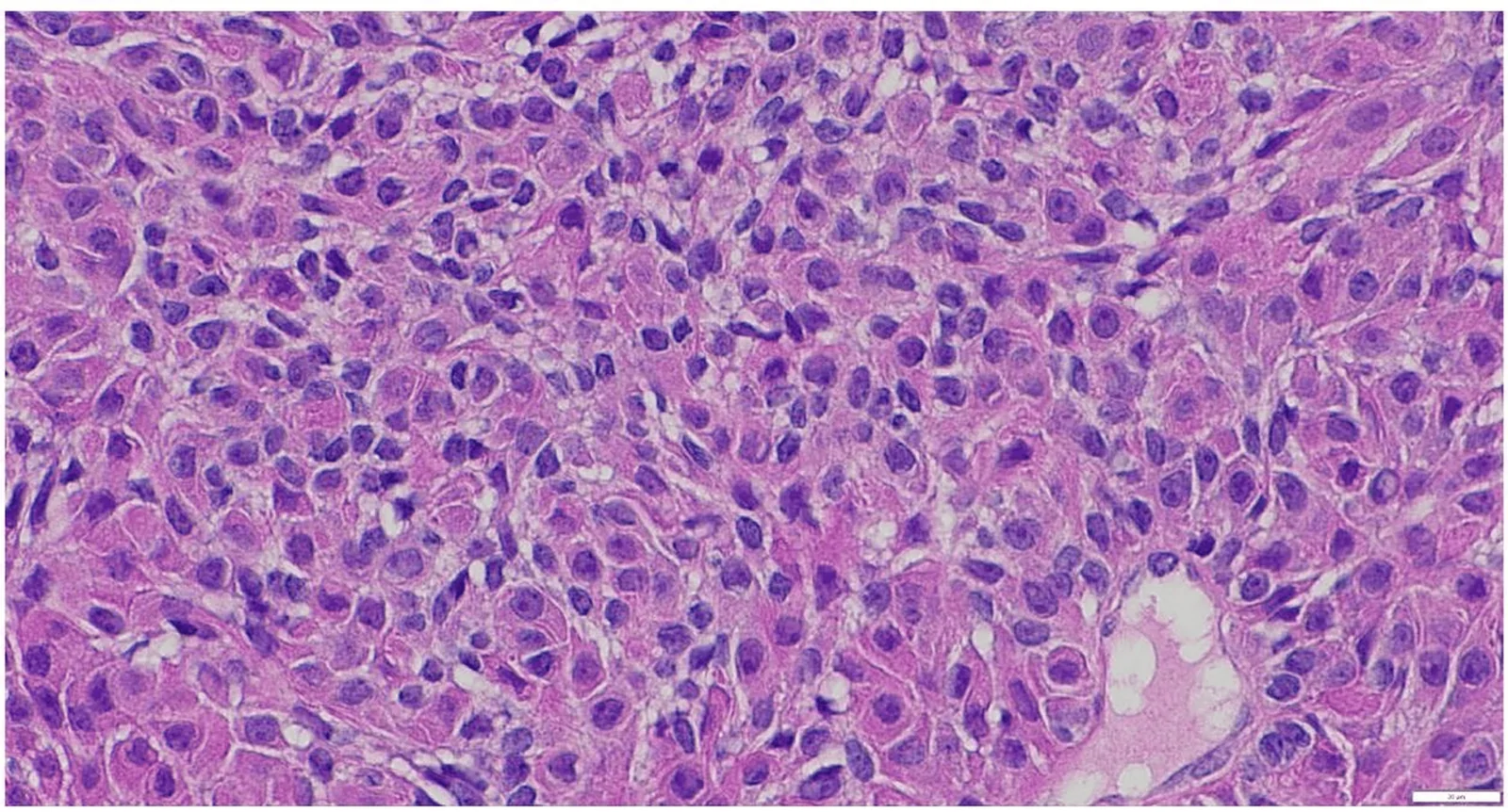
High-power magnification of the lesion shows predominantly polygonal cells with eosinophilic cytoplasm and clear cell change. (H&E, scale bar = 20 μm)

The final diagnosis was solid and cystic hidradenoma. Given the negative margins, low risk of recurrence, and benign nature of this tumor type, clinical observation was planned.

The patient was followed up for one year postoperatively, with no clinical evidence of local recurrence. She subsequently developed a hypertrophic scar at the surgical site, for which intralesional corticosteroid injection was offered; the patient elected to defer treatment at that time. Timeline of presentation, evaluation, and management is presented in Table 1.

**Table 1:** Timeline of presentation, evaluation, and management.

Event	Details
Symptom onset	Left forearm nodule first noted by patient; gradual enlargement over approximately two years prior to presentation
Initial presentation	Evaluation for a tender, mobile, irregularly nodular lesion (3 x 2 cm) on the radial volar forearm; hidradenoma not initially considered
Preoperative imaging	Ultrasonography performed, demonstrating a 3.6 x 2.0 x 1.4 cm subcutaneous mass with mixed solid and cystic components
Preoperative decision-making	Ultrasound recommended given proximity to important anatomical structures; further imaging (CT/MRI) vs. direct excision discussed with patient, who elected excision
Surgical intervention	Elliptical excision performed under local anesthesia with local flap closure
Pathologic diagnosis	Solid-cystic hidradenoma confirmed; margins reported negative
Surgical margins	Excision limited to subcutaneous plane (deep fascia not taken); no predetermined margin planned, entire mass excised (~4 x 3 cm specimen intraoperatively); closest microscopic margin 2 mm (0.2 cm)
Postoperative follow-up	Followed for one year with no clinical evidence of local recurrence; developed a hypertrophic scar at the surgical site, for which intralesional corticosteroid injection was offered and deferred by the patient

## Discussion

Hidradenomas are uncommon, benign, slow-growing adnexal tumors that arise from either eccrine or apocrine sweat glands and typically range from 1 to 3 cm in size [1]. The coexistence of solid and cystic components, as seen in our patient, is not itself an atypical finding; it is an inherent, defining characteristic of the solid-cystic (clear-cell) subtype [1]. Demographically, hidradenomas most commonly occur in middle-aged adults with a slight female predominance [1]. Our patient, a 41-year-old woman, aligns with this typical age and sex distribution, although the size of her tumor was at the larger end of the reported range.

These tumors can arise anywhere on the body but are most frequently reported on the head, neck, and trunk [1]. The principal source of rarity in this case is therefore anatomical rather than histological: comparatively few hidradenomas of any subtype have been documented in the extremities [2,4,5], and the forearm in particular has been reported only rarely. Based on a literature review conducted through PubMed and Google Scholar, we identified only two prior hidradenoma-family tumors reported at this specific site: a solid-cystic (clear-cell) hidradenoma in a five-year-old boy [4] and a poroid hidradenoma, a related but histologically distinct, eccrine-differentiated variant, in an adult [5]. We did not identify a prior adult case of solid-cystic (clear-cell) hidradenoma at the forearm. Among the cases identified in this non-systematic review, our case therefore appears to be the first such case in an adult patient, adding to a very small body of literature on forearm hidradenomas across all subtypes [4,5]. Other subtypes, including additional poroid and nodular variants, have also been described in atypical extremity locations more broadly [5-9]; taken together, this pattern reinforces that hidradenoma should remain in the differential diagnosis of any subcutaneous nodule, regardless of anatomical site.

Beyond its rarity, the anatomical location in this case carries direct clinical significance. Extremity lesions, and forearm lesions in particular, are more likely to be mistaken for other entities that carry different management implications, most notably dermatofibrosarcoma protuberans, which requires wide local excision with margin control rather than the simple excision appropriate for a benign hidradenoma. A delayed or incorrect diagnosis in this location could therefore lead to either under-treatment of a malignant process or unnecessarily aggressive management of a benign one. In addition, forearm lesions frequently require reconstructive closure, as in our patient, making accurate preoperative or intraoperative assessment relevant to surgical planning. These considerations underscore why hidradenoma should be actively considered, not simply included as a footnote, in the differential diagnosis of nodular lesions at atypical extremity sites.

The initial differential diagnosis in this case included epidermal inclusion cyst, pilomatricoma, dermatofibroma, and dermatofibrosarcoma protuberans, reflecting the nonspecific clinical appearance of hidradenoma. Epidermal inclusion cysts typically demonstrate a central punctum and are more firmly fixed to the overlying dermis, whereas hidradenomas are usually mobile and lack a punctum, as in our patient. Pilomatricoma classically presents as a firm, often calcified nodule that produces a characteristic tenting or faceted appearance of the overlying skin on stretch and is more common in children and young adults. Dermatofibromas are typically smaller and firmer, and characteristically dimple inward on lateral compression.

Dermatofibrosarcoma protuberans, the most clinically important diagnosis to exclude given its potential for local invasion, typically presents as a slowly enlarging, indurated plaque or nodule that may become adherent to surrounding structures and shows infiltrative margins with a storiform growth pattern on histology, in contrast to the well-circumscribed, encapsulated appearance typical of hidradenoma. The mobile, well-circumscribed nature of our patient's lesion, together with its mixed solid-cystic appearance on ultrasonography, was more consistent with a benign adnexal process, although, as detailed above, histopathologic confirmation after excision remained necessary to definitively exclude malignancy.

Preoperative imaging findings in hidradenoma are nonspecific but often demonstrate a well-circumscribed mass with mixed cystic and solid components on ultrasonography, consistent with our patient's imaging and with prior reported cases [10]. While such findings can help narrow the differential diagnosis, histopathologic confirmation remains necessary, since imaging alone cannot reliably distinguish hidradenoma from other cystic or solid adnexal and soft-tissue lesions.

Although malignant transformation of hidradenoma is rare, it has been described and underscores the importance of complete excision with negative margins and appropriate clinical follow-up, even in cases judged to be low risk [11]. In our patient, negative margins and the benign histopathology supported a plan of clinical observation without adjuvant therapy.

This case has several limitations. No preoperative tissue diagnosis was obtained, and the decision to proceed directly to excision, discussed with and elected by the patient after considering additional imaging, relied on nonspecific imaging findings rather than histologic confirmation. The histopathologic assessment was similarly based on routine morphology alone, without immunohistochemistry or an itemized description of features such as mitotic activity or invasion beyond what is reported above; while the final diagnosis was benign, a more granular histologic characterization was not available for this report. Although the patient was followed for one year without evidence of recurrence, longer-term surveillance would provide additional reassurance given the limited data on recurrence risk at this anatomical site. As with any single case report, this report cannot establish the true incidence of hidradenoma at the forearm; our characterization of this location as rare reflects a non-systematic review of the published literature rather than an exhaustive or systematic search and should be interpreted accordingly.

## Conclusions

This case illustrates an unusual presentation of solid-cystic hidradenoma on the volar forearm. The clinical significance of this case lies primarily in its anatomical location rather than its solid-cystic morphology: forearm involvement has been reported only rarely and, to our knowledge, never before in an adult with this specific subtype. This rarity, combined with the tumor's nonspecific clinical presentation and its clinical overlap with more consequential diagnoses such as dermatofibrosarcoma protuberans, contributed to a broad initial differential diagnosis, and histopathologic confirmation was required to establish the diagnosis. This case highlights the importance of keeping hidradenoma as a differential diagnostic consideration for subcutaneous nodules arising on the extremities, an anatomical distribution for which comparable adult cases remain limited.
